# Detail Preserved Surface Reconstruction from Point Cloud

**DOI:** 10.3390/s19061278

**Published:** 2019-03-13

**Authors:** Yang Zhou, Shuhan Shen, Zhanyi Hu

**Affiliations:** 1 National Laboratory of Pattern Recognition, Institute of Automation, Chinese Academy of Sciences, Beijing 100190, China; yang.zhou@nlpr.ia.ac.cn (Y.Z.); huzy@nlpr.ia.ac.cn (Z.H.); 2 School of Artificial Intelligence, University of Chinese Academy of Sciences, Beijing 100049, China

**Keywords:** computer vision, 3D reconstruction, point cloud

## Abstract

In this paper, we put forward a new method for surface reconstruction from image-based point clouds. In particular, we introduce a new visibility model for each line of sight to preserve scene details without decreasing the noise filtering ability. To make the proposed method suitable for point clouds with heavy noise, we introduce a new likelihood energy term to the total energy of the binary labeling problem of Delaunay tetrahedra, and we give its *s*-*t* graph implementation. Besides, we further improve the performance of the proposed method with the dense visibility technique, which helps to keep the object edge sharp. The experimental result shows that the proposed method rivalled the state-of-the-art methods in terms of accuracy and completeness, and performed better with reference to detail preservation.

## 1. Introduction

Image-based scene reconstruction is a fundamental problem in Computer Vision. It has many practical applications in fields such as entertainment industry, robotics, cultural heritage digitalization and geographic systems. Image-based scene reconstruction has been studied for decades due to its low cost data acquisition and various usages. In recent years, researchers have made tremendous progress in this field. As far as small objects under controlled conditions are concerned, the performance of current scene reconstruction methods could achieve results comparable to those generated by laser scans or structured-light based methods [1,2]. However, when it comes to large scale scenes with multi-scale objects, current reconstruction methods have some problems with the completeness and accuracy, especially when concerning scene details [3].

**Figure 1:**
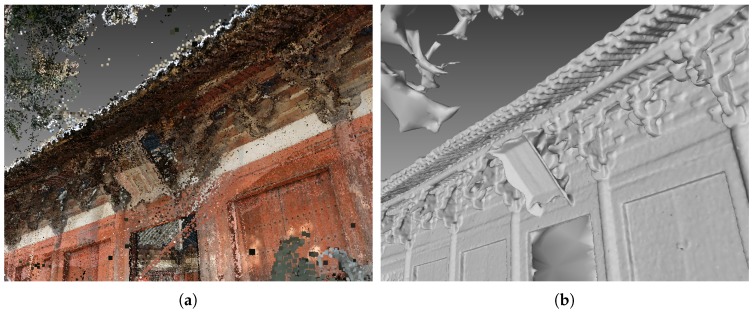
An example of the application of the proposed method in the field of cultural heritage digitalization, (**a**) point cloud; (**b**) mesh.

Scene details such as small scale objects and object edges are an essential part of scene surfaces. Figure 1 shows an example of preserving scene details in reconstructing an ancient Chinese architecture. In general, cultural heritage digitalization projects, representing scene details such as the brackets in Figure 1, are among the most important tasks. The point cloud representation is often redundant and noisy, and the mesh representation is concise but it sometimes lose some information. Therefore, preserving scene details in reconstructing multi-scale scenes has been a difficult problem in surface reconstruction. The existing surface reconstruction methods [4,5,6,7] either ignore the scene details or rely on further refinement to restore them. Firstly, this is because, compared with noise, the supportive points in such part of the scene are sparse, making it difficult to distinguish true surface points from false ones. Secondly, the visibility models and associated parameters employed in existing methods are not particularly suitable for large scale ranges, where scene details are usually compromised for overall accuracy and completeness. While the first case seems to be unsolvable due to the lack of sufficient information, in this work, we focus on the second case. In particular, we extend the work of our conference paper [8], and suggest a new method with a new visibility model for surface reconstruction.

In many previous surface reconstruction methods [4,5,6,9,10,11,12,13], visibility information that records a 3D point is seen by the views used to help to generate accurate surface meshes. To use the visibility information, assumptions of the visibility model are made so that the space between camera center and the 3D point is free-space and the space behind the point along the line of sight is full-space. However, the points are often contaminated with noise and the full-space scales are often hard to determinate. To preserve scene details without decreasing the noise filtering ability, we propose a new visibility model with error tolerance and adaptive end weights. We also introduce a new likelihood energy representing the punishment of wrongly classifying a part of space as free-space or full-space, which helps to improve the ability of the proposed method to efficiently filter noise. Moreover, we further improve the performance of the proposed method with the dense visibility technique, which helps to keep the object edge sharp. Experimental results show that the proposed method rivals the state-of-the-art methods in terms of accuracy and completeness, and performs better with reference to detail preservation.

## 2. Related Work

In recent years, various works have been done to advance the image-based scene reconstruction. Referring to small objects, silhouette based methods [14,15,16,17,18] are proposed. The silhouettes provide proper bounds for the objects, which help reduce the computing cost and yield a good model for the scene. However, good silhouettes rely on effective image segmentation, which remains a difficult task. Furthermore, silhouettes can hardly be used for large scale scenes. Volumetric methods such as space carving [19,20,21], level sets [22,23] and volumetric graph cut [9,10,11] often yield good results for small objects. However, in the case of large scale scenes, the computational and memory costs increase rapidly as scene scale grows. Consequently, this makes them unsuitable for large scale scene reconstruction. Vogiatzis et al. [9] proposed a volumetric graph cut based method to reconstruct an object by labeling voxels as inside or outside, in which a photo-consistency term is introduced to enhance the final result. Tran and Davis [10] and Lempitsky et al. [11] also exploited the same idea, the former by adding predetermined locations of possible surface as surface constraints and the latter by estimating visibility based on position and orientation of local surface patches, then optimizing on a CW-complex.

As far as outdoor scenes, uncontrollable imaging conditions and multiple scale structures make it hard to reconstruct scene surfaces. A common process of reconstructing large scale scenes is to generate the dense point cloud from calibrated images first, and then extract the scene surface. The dense point cloud can be generated through the depth fusion method [24,25,26,27,28] or through the feature expansion methods [29]. In depth-map fusion methods, depth-maps are usually computed independently, and then merged into one point cloud; in feature expansion methods instead, the sparse point cloud is generated first, and then expanded to points near the seed points. Usually, the depth-map fusion based methods yield a relatively denser but noisier point cloud. Once the dense point cloud is generated, scene surface can be reconstructed through poisson surface reconstruction [30] or through graph cut based methods [4,5,12,13]. In [12], firstly the dense point cloud is used to generate Delaunay tetrahedra, then a visibility model is introduced to weight the facets in Delaunay tetrahedra, and finally the inside–outside binary labeling of tetrahedra is solved and the surface is extracted; it consists of triangles between tetrahedra with different labels. The basic assumption of the visibility model in [12] is that the space between camera center and 3D point is free-space, while the space behind 3D point is full-space. Then, this typical visibility model is promoted by a refined version in [13], namely soft-visibility, to cope with noisy point clouds. Apart from using dense point clouds, Bódis-Szomorú et al. [31] also proposed a method to produce a surface mesh by fitting the meshes reconstructed by single views and a sparse point cloud. The method is parallelizable and the quality of the final meshes is comparable to that of a state-of-the-art pipeline [32].

Besides the point-cloud based methods, large scale scene reconstruction can also be achieved through volume-based methods. Häne et al. [6,33] proposed a method for scene reconstruction and object classification. The scene space is represented by voxels and each one is given a class label. The accuracy of the classification relies on the decision tree, which is made up of labeled images. The extensive works in [34,35] reduce the heavy memory cost of the method in [33] by introducing octree structure and block scheme, respectively. Savinov et al. [36] exploited an idea similar to that in [6,33], and used full multilabel ray potential and continuously inspired anisotropic surface regularization together to yield 3D semantic models. Ummenhofer and Brox [37] proposed a method that is capable of handling a billion points. This method uses an octree structure to manage the points and then reconstruct the scene using a level-set alike method.

In this paper, we extend the work of our conference paper [8], follow the idea in [5,13] and exploit the visibility model for the line of sight in the binary labeling problem of Delaunay tetrahedra. The main contributions of the proposed method are: (1) two new visibility models for the visibility information of the points on the vertices of Delaunay tetrahedra and inside them, respectively; and (2) the dense visibility technique, which exploits the visibility information of unmerged points for better performance of preserving scene details. Experimental comparison results show that the proposed method rivals the state-of-the-art methods [5,24,27,29,38] in terms of accuracy and completeness, and performs better in detail preservation.

## 3. Visibility Models and Energies

**Figure 2:**
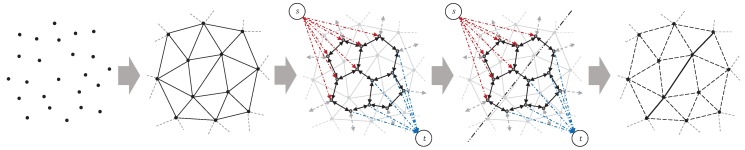
Pipeline of the proposed method in 2D. From left to right are the input point cloud, Delaunay tetrahedra, the *s*-*t* graph, the energy minimization result and the final surface mesh.

The pipeline of the proposed method is shown in Figure 2. The input of the proposed method is a dense point cloud generated by multi-view stereo (MVS) methods. Each point in the point cloud is attached with the visibility information recording that from which views the point is seen. Next, Delaunay tetrahedra are constructed from given input point cloud, and the scene reconstruction problem is formulated as a binary labeling problem to label tetrahedra as inside or outside. Then, an *s*-*t* graph is constructed with tetrahedra as the vertices and facets of tetrahedra as the edges. Finally, by minimizing the energy defined on the *s*-*t* graph, the vertices are separated into two parts, i.e., inside and outside, and the scene surface is extracted which consists of triangle facets lying between tetrahedra with different labels. The key issue of this process is to find a proper energy. In doing so, we introduce a new visibility model to formulate the energy of the binary labeling problem. In the following subsections, we detail the visibility models in [12,13] and our new one. Before that, we give the meaning of the symbols used in this work in Table 1 for better understanding.

### 3.1. Existing Visibility Models

**Figure 3:**
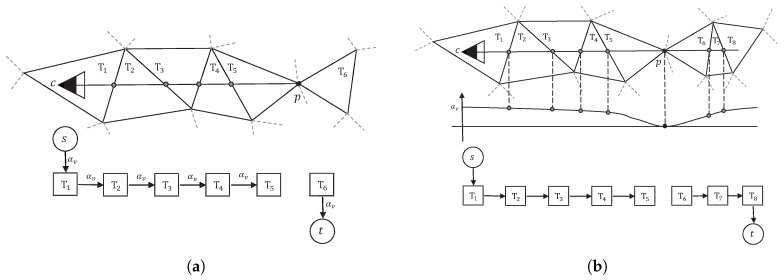
Typical visibility model and soft visibility model in 2D. (**a**) Typical visibility model; and (**b**) soft visibility model for a single line of sight *v*; how to assign weight (energy) to the tetrahedron containing camera center, the end tetrahedron and the facets intersected by (c,p) or its extension are shown.

The typical visibility model [12] assumes that, for each line of sight *v*, the space between camera center *c* and point *p* is free-space; the space behind point *p* along the line of sight is full-space. For the Delaunay tetrahedra of the point cloud, the tetrahedra intersected by segment (c,p) should be labeled as outside; the tetrahedron right behind the point *p* should be labeled as inside. For example, in Figure 3a, according to the above model, tetrahedra $T_{1}$–$T_{5}$ should be labeled as outside, while tetrahedron $T_{6}$ should be labeled as inside.

For a single line of sight, the above label assignment is desirable. While taking all lines of sight into consideration, some surface part might not be handled properly, for example, that between $T_{2}$ and $T_{3}$ in Figure 3a. This scenario violates the label assignment principle described above. To punish the conflicts, the facets intersected by a line of sight are given weights (energy) of $W(l_{T_{i}},l_{T_{j}})$. Similarly, weights for punishing bad label assignments of the first tetrahedron and last tetrahedron are $D(l_{T_{1}})$ and $D(l_{T_{N_{v}+1}})$, respectively. Therefore, the visibility energy is the sum of the penalties of all the bad label assignments in all the lines of sight, as

(1) $$E_{vis}^{typical}=\sum_{v\in V}\left[D\left(l_{T_{1}}\right)+\sum_{i=1}^{N_{v}-1}W\left(l_{T_{i}},l_{T_{i+1}}\right)+D\left(l_{T_{N_{v}+1}}\right)\right]$$

where

$$D\left(l_{T_{1}}\right)=\left\{\begin{array}{cc} \alpha_{v} & \mathrm{if}\;l_{T_{1}}=1\\ 0 & \mathrm{otherwise} \end{array}\right.,\;D\left(l_{T_{N_{v}+1}}\right)=\left\{\begin{array}{cc} \alpha_{v} & \mathrm{if}\;l_{T_{N_{v}+1}}=0\\ 0 & \mathrm{otherwise} \end{array}\right.,\;W\left(l_{T_{i}},l_{T_{j}}\right)=\left\{\begin{array}{cc} \alpha_{v} & \mathrm{if}\;l_{T_{i}}=0\;\wedge \;l_{T_{j}}=1\\ 0 & \mathrm{otherwise} \end{array}\right.$$

where V denotes the visibility information set, containing all lines of sight; $N_{v}$ is the number of tetrahedra intersected by a single line of sight *v*, indexed from the camera center *c* to the point *p*; $N_{v}+1$ denotes the tetrahedron behind the point *p*; $l_{T}$ is the label of tetrahedron *T*, with 1 stands for inside and 0 outside; $\alpha_{v}$ is the weight of a line of sight *v*, which can be the photo consistency score of point *p* in current view.

The typical visibility model as well as the energy formulation in Equation (1) described above is effective in most cases, but it has several flaws in relation to dense and noisy point clouds [13]. The surfaces reconstructed by [12] tend to be overly complex, with bumps on the surface and handles inside the model. One possible solution to these problems is the soft visibility proposed in [13], as shown in Figure 3b. In the soft visibility model, the basic assumption is similar to the previous typical visibility model. However, the edge weights are multiplied by a weight factor $\left(1-e^{-d^{2}/2\sigma^{2}}\right)$, in which *d* represents the distance between the point *p* and the intersecting point. In addition, the end tetrahedron in the soft visibility model is shifted to a distance of 3σ along the line of sight.

### 3.2. Our Proposed Visibility Model

Although the soft visibility model is effective to filter noise points and helps to yield visually smoothed models, it sometimes performs poorly in preserving details, especially in a large scene containing some relatively small scale objects (see Experimental Results). According to our observations, this happens mainly because of the improperly chosen relaxation parameter σ and the strong constraint imposed on the end of line of sight in the tetrahedron kσ from the point *p* along the line of sight. In some cases, such end tetrahedra would be free-space even though the point *p* is a true surface point.

**Figure 4:**
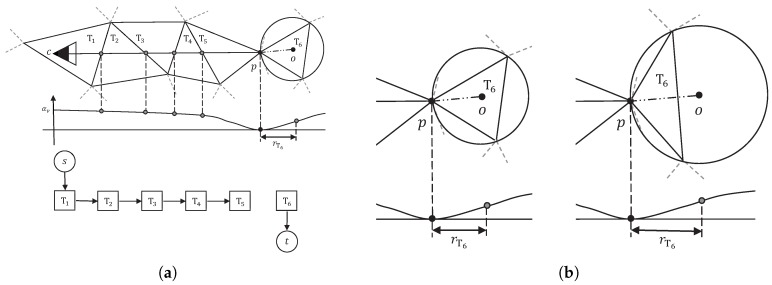
Our visibility model and end tetrahedra comparison in 2D. (**a**) In our visibility model, for a single line of sight *v*, how to assign weight (energy) to the tetrahedron containing camera center, the end tetrahedron and the facets intersected by (c,p) is shown. (**b**) From left to right: Typical end tetrahedron of noise points and that of true surface points on densely sampled surfaces.

To balance noise filtering and detail preserving, we propose a new visibility model, which is shown in Figure 4a. In our visibility model, we also use the relaxed visibility constraints in the soft visibility model in the space between the camera center *c* and the point *p*, i.e., the weight factor $\left(1-e^{-d^{2}/2\sigma^{2}}\right)$ is kept to ensure that the final model is not overly complex. Then, we set the end of line of sight in the tetrahedron just right behind the point *p*, to avoid the wrong end in the soft visibility model in the case of small scale objects. To determine the weight of the *t*-edge of the end tetrahedron, we compare the end tetrahedra of noisy points and true surface points on datasets with quasi-truth. Figure 4b shows a typical end tetrahedron of noisy points and that of true surface points on densely sampled surfaces in 2D space. Noise points tend to appear in somewhere a bit away from true surface, which makes the end tetrahedra (triangles in 2D) thin and long, and true surface points are often surrounded by other true surface points, which makes their end tetrahedra flat and wide. Based on the above observations, we set a weight of $\alpha_{v}\left(1-e^{-r^{2}/2\sigma^{2}}\right)$ to the *t*-edge of the end tetrahedron, where *r* is the radius of the circumsphere of the end tetrahedron.

With our new visibility model, our visibility energy is formulated as

(2) $$E_{vis}=\sum_{v\in V}\left[D\left(l_{T_{1}}\right)+\sum_{i=1}^{N_{v}-1}W\left(l_{T_{i}},l_{T_{i+1}}\right)+D\left(l_{T_{N_{v}+1}}\right)\right]$$

where

$$D\left(l_{T_{1}}\right)=\left\{\begin{array}{cc} \alpha_{v} & \mathrm{if}\;l_{T_{1}}=1\\ 0 & \mathrm{otherwise} \end{array}\right.,\;D\left(l_{T_{N_{v}+1}}\right)=\left\{\begin{array}{cc} \alpha_{v}\left(1-e^{-r^{2}/2\sigma^{2}}\right) & \mathrm{if}\;l_{T_{N_{v}+1}}=0\\ 0 & \mathrm{otherwise} \end{array}\right.,$$

$$W\left(l_{T_{i}},l_{T_{j}}\right)=\left\{\begin{array}{cc} \alpha_{v}\left(1-e^{-d^{2}/2\sigma^{2}}\right) & \mathrm{if}\;l_{T_{i}}=0\;\wedge \;l_{T_{j}}=1\\ 0 & \mathrm{otherwise} \end{array}\right.$$

Instead of setting the tolerance parameter σ to a constant manually, we set σ adaptively within 0.5–1% of the length of line of sight in our visibility model. The underlying reason is that, typically, in depth-map fusion methods, the error bound used to filter bad correspondences while generating dense point clouds is adaptively set to 0.5–1% of the depth of the point in the current view, as in [28]. This gives each point a confidence interval along the line of sight.

## 4. Likelihood Energy for Efficient Noise Filtering

**Figure 5:**
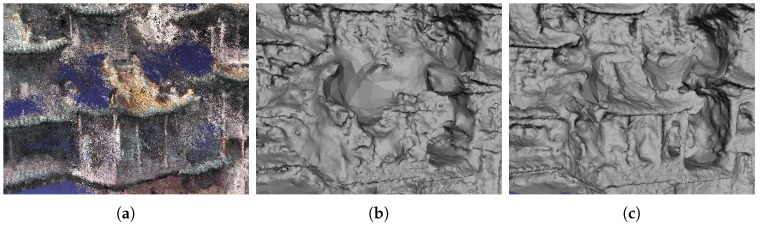
Surface reconstruction without and with the likelihood energy. From left to right: (**a**) point cloud with heavy noise; (**b**) reconstructed meshes without; and (**c**) with the likelihood energy.

In both the typical visibility model and our proposed one, the end of each line of sight is set in the tetrahedra right behind the point *p*. This practice sometimes could weaken the ability of noise filtering. When the surface is sampled very densely, unexpected handles could appear inside the model [13], or part of the surface could fail to be reconstructed, as shown in Figure 5. This is mainly due to the unbalanced links of *s*-edges and *t*-edges in the *s*-*t* graph, i.e., the *s*-edges are too strong to be cut as the camera centers are consistent for all lines of sight, while the *t*-edges are weak because their weights are scattered by the varying locations of the points. The noisier the point cloud is, the greater the gap between *s*-edges and *t*-edges becomes.

### 4.1. Likelihood Energy

To solve these problems, we introduce a likelihood energy $E_{like}$ to the total energy of the binary labeling problem. $E_{like}$ is defined as

(3) $$E_{like}=\sum_{i=1}^{N}D\left(l_{T_{i}}\right),\;\mathrm{where}\;D\left(l_{T_{i}}\right)=\left\{\begin{array}{cc} U_{out}\left(T_{i}\right) & \mathrm{if}\;l_{T_{i}}=1\\ U_{in}\left(T_{i}\right) & \mathrm{otherwise} \end{array}\right.$$

where *N* is the total number of Delaunay tetrahedra. $E_{like}$ measures the penalties of a wrong label assignment. For each tetrahedron, it is attached with two attributes that describe how likely it is to be outside or inside. If it is mistakenly labeled, a penalty is introduced, i.e., $U_{out}\left(T_{i}\right)$ or $U_{in}\left(T_{i}\right)$.

To evaluate the likelihood of the label assignment of a tetrahedron, we employ the measure *free-space support* [5], which is used to measure the emptiness of a compact space. For each line of sight *v* that intersects tetrahedra *T*, it contributes to the emptiness of $T_{i}$, thus increasing the probability of *T* to be outside. The free-space support f(T) of tetrahedron *T* is computed as

(4) $$f\left(T\right)=\sum_{v\in V_{T}}\alpha_{v},\;\mathrm{with}\;V_{T}=\left\{v|v\cap T\neq \emptyset \right\}$$

where $V_{T}$ is the set of lines of sight *v* that intersect with tetrahedron *T*. To evaluate f(T) to adequately describe the likelihood energy, we set $U_{out}\left(T\right)=\lambda f\left(T\right)$ and $U_{in}\left(T\right)=\lambda \left(\beta -f\left(T\right)\right)$, where λ is a constant used to scale the range of f(T), and β is a constant greater than f(T) for all tetrahedra *T*.

### 4.2. Implementation of the Likelihood Energy

For the likelihood term $E_{like}$, we link two edges for each vertex *i* in *s*-*t* graph. One is from source *s* to vertex *i*, with weight $U_{out}\left(T_{i}\right)$; the other is from vertex *i* to sink *t*, with weight $U_{in}\left(T_{i}\right)$. However, to reduce the complexity of the graph, we cross out the *s*-edges of all vertices, and only link the vertices to sink *t* whose correspondent tetrahedra have lower f(T) than the 75th percentile of all f(T)s. Since some of the vertices in *s*-*t* graph have a heavy-weight edge linked to source *s*, we rely on the visibility constraints to label truly outside tetrahedra, instead of linking them with extra *s*-edges.

**Figure 6:**
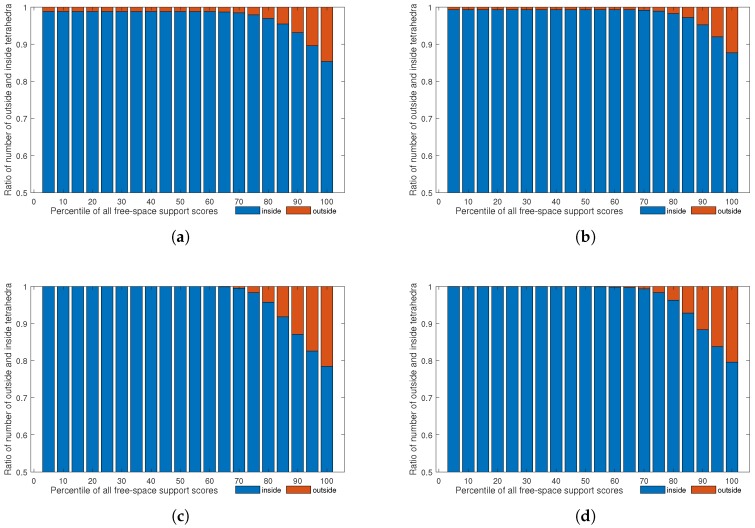
Free-space support analysis. The four graphs show the ratios of number of outside and inside tetrahedra in different percentiles of all free-space support scores. The four datasets are: (**a**) Fountain-P11 [3]; (**b**) Herz-Jesu-P8 [3]; (**c**) Temple-P312 [1]; and (**d**) scan23-P49 [2].

**Figure 7:**
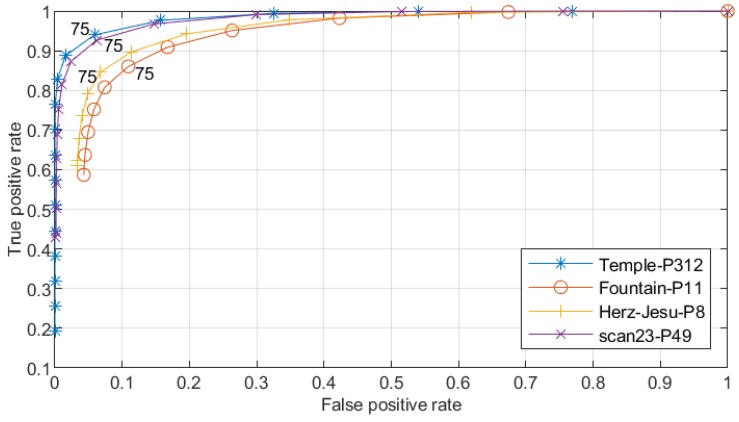
Free-space support threshold evaluation. The true positive rates and false positive rates in the same four datasets as in Figure 6 are evaluated by setting different free-space support thresholds.

Note that the free space support threshold of 75th percentile was empirically set, as shown in Figure 6 and Figure 7. We generated dense point clouds of four datasets, and applyiedDelaunay tetrahedralization to them. The four datasets were Fountain-P11 [3], Herz-Jesu-P8 [3], Temple-P312 [1] and scan23-P49 [2]. Then, we labeled the tetrahedra with method described in [5], and took the result as the quasi-truth. Finally, we evaluated the ratios of number of outside and inside tetrahedra in different proportions of all free-space support scores, as shown in Figure 6. In Figure 6, we can see that, generally, tetrahedra with lower f(T) (f(T) lower than the 75th percentile of all free-space support scores) had a higher probability to be truly inside. As shown in Figure 7, the true positive rates and false positive rates with different free-space support thresholds were evaluated. It is noteworthy that, when the free-space support threshold was set as the 75th percentile of each dataset, both true positive rate and false positive rate were reasonable. Therefore, we only linked those tetrahedra to sink *t* whose free-space support was lower than 75th percentile of all free-space support scores. In Figure 5, we can see that the likelihood energy is helpful for filtering noise.

## 5. Surface Reconstruction with Energy Minimization

With the likelihood energy and the proposed visibility model, the total energy of the binary labeling problem of the Delaunay tetrahedra is formulated as

(5) $$E_{total}=E_{vis}+\lambda_{like}E_{like}+\lambda_{qual}E_{qual}$$

where $\lambda_{like}$ and $\lambda_{qual}$ are two constant balancing factors; $E_{vis}$ and $E_{like}$ are defined in Equations (2) and (3); and $E_{qual}$ is the surface quality energy introduced in [13] as

(6) $$E_{qual}=\sum_{f}w_{f}$$

where

$$w_{f}=1-\min\left\{\cos\left(\phi \right),\cos\left(\psi \right)\right\},\;\mathrm{if}\;l_{T_{1}^{f}}\neq l_{T_{2}^{f}}$$

The total energy $E_{total}$ defined in Equation (5) could be represented as an *s*-*t* graph and minimized by the Maxflow/Mincut algorithm [39]. In $E_{total}$, the graph construction for the visibility energy $E_{vis}$ and the surface quality energy $E_{qual}$ is straightforward, and the likelihood $E_{like}$ is implemented as described in Section 4.2. Then, the energy minimization problem is solved using the Maxflow/Mincut algorithm on the *s*-*t* graph, and the optimal label assignment of Delaunay tetrahedra is yielded. Ultimately, a triangle mesh, which consists of triangles lying between tetrahedra with different labels, is extracted. A further optional refinement can be applied as described in [7].

## 6. Dense Visibility for Edge Preservation

**Figure 8:**
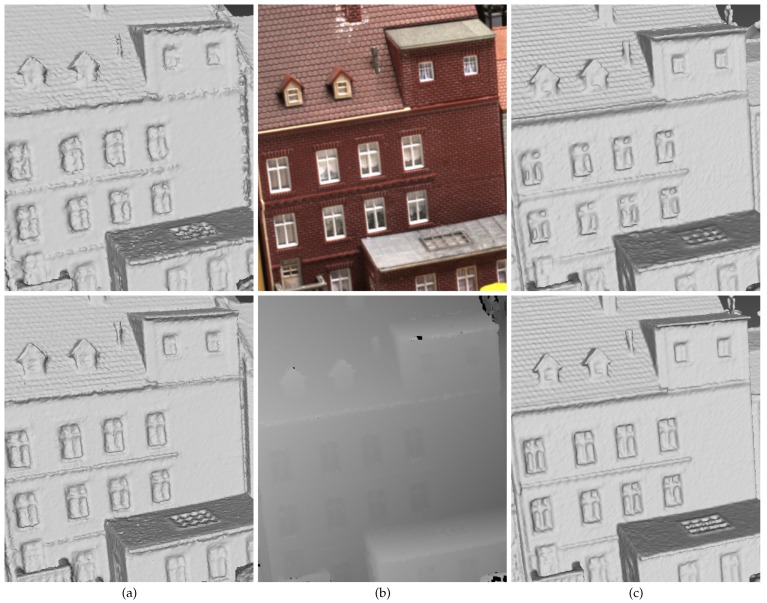
Object edges in the reconstructed meshes, the original image and the depth map. From left to right: (**a**) the object edges in surface meshes reconstructed by the method in [5] (top) and our method (bottom) without the dense visibility technique; (**b**) the original image and the corresponding depth map with a similar view point; and (**c**) the object edges in surface meshes reconstructed by the method in [5] (top) and our method (bottom) with the dense visibility technique.

**Figure 9:**
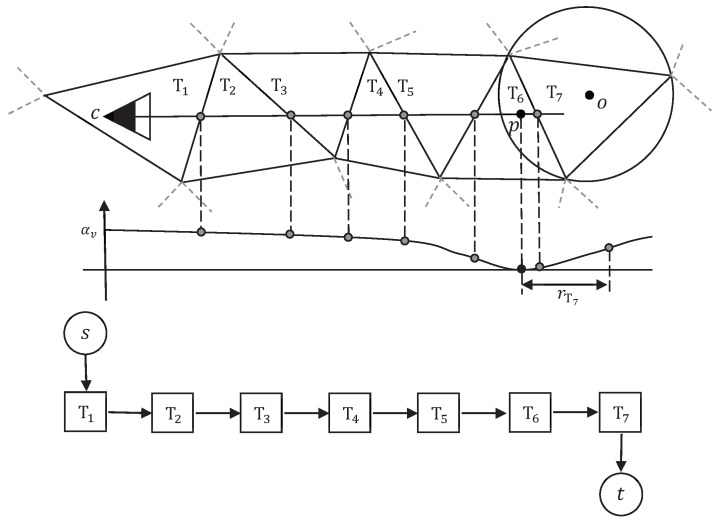
The modified version of our visibility model in 2D. In this visibility model, for a single line of sight *v*, how to assign weight (energy) to the tetrahedron containing camera center, the end tetrahedron and the facets intersected by (c,p) or its extension is shown.

Although the proposed visibility model in Figure 4 and the energy formulation in Equation (5) are carefully designed for preserving the scene details, the object edges sometimes still appear to be inaccurate concerning bumps and dents. When referring to the original depth maps, the depths of object edges are quite smooth. This scenario is shown in Figure 8. Figure 8a,b shows the object edges in 3D meshes reconstructed by the method in [5] and the proposed method described in the previous sections, as well as the original image and the corresponding depth map with a similar view point. We can easily see that the object edges in the reconstructed meshes failed to keep the smoothness as in the depth map. This could be due to the error of either point locations or visibility information which is introduced in the depth map fusion process. To circumvent such problem, instead of fusing the depths in matched cameras, we generate the dense point cloud simply by joining all of the 3D points recovered with depth maps and camera parameters. This could result in a much denser and more redundant point cloud, but it also contains more useful information for better surface reconstruction. To alleviate the memory and the computational cost, points are sampled and then Delaunay tetrahedra are constructed from the sampled point cloud. Instead of discarding those points unused for tetrahedralization and their visibility information, we apply a modified version of our visibility model to use their visibility information, as shown in Figure 9. To keep the ability to filter noise and select true surface points, we keep most of the visibility model in Figure 4 along the line of sight and only modify the part near the 3D point. The difference between the visibility models in Figure 9 and the one in Figure 4 is that, for a point *p* that lies in a tetrahedron, the end of the line of sight is set in the tetrahedron right behind the one that contains *p*, and the facet between them is punished with a weight multiplied by the same weight factor as in the soft visibility model. In this way, we keep the end of the line of sight close to the 3D point and the tolerance to noise. In Figure 8c, we show the surface meshes containing object edges reconstructed by the method in [5] and the proposed method with the dense visibility technique. We can infer from the results in Figure 8 that the dense visibility technique is helpful for preserving object edges.

## 7. Experimental Results

In our experiments, the input dense point cloud was generated from images with the open source library OpenMVG (http://imagine.enpc.fr/~moulonp/openMVG/) and OpenMVS (http://cdcseacave.github.io/openMVS/). Sparse point cloud was generated by OpenMVG, then densified with OpenMVS. Delaunay tetrahedralization was computed using CGAL (http://www.cgal.org/) library. Maxflow/Mincut algorithm [39] aws used. The proposed method was tested on public benchmark MVS dataset [2] and Tanks and Temples dataset [40].

**Figure 10:**
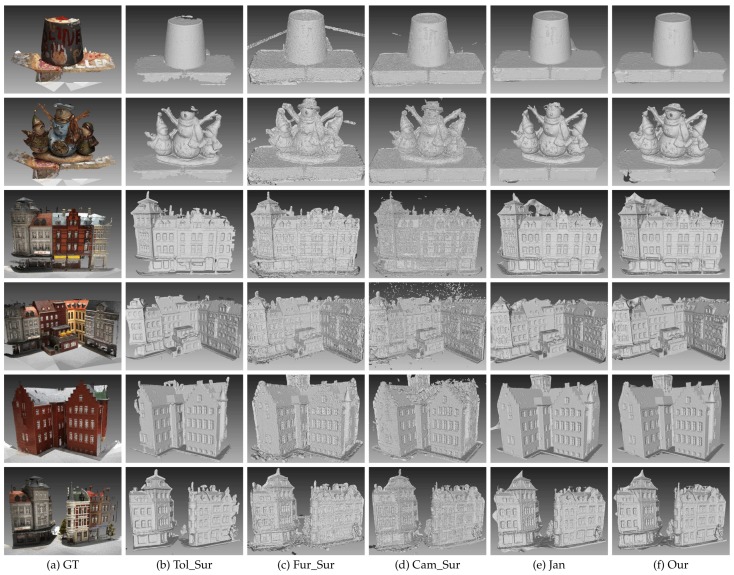
Result of the five methods on MVS dataset [2]. GT is the reference model; Tol is Tola et al. [27]; Fur is Furukawa and Ponce [29]; Cam is Campbell et al. [24]; Jan is Jancosek and Pajdla [5]; Our is the proposed method; and ∗_Sur is the surface generated by poisson surface reconstruction method [30].

**Figure 11:**
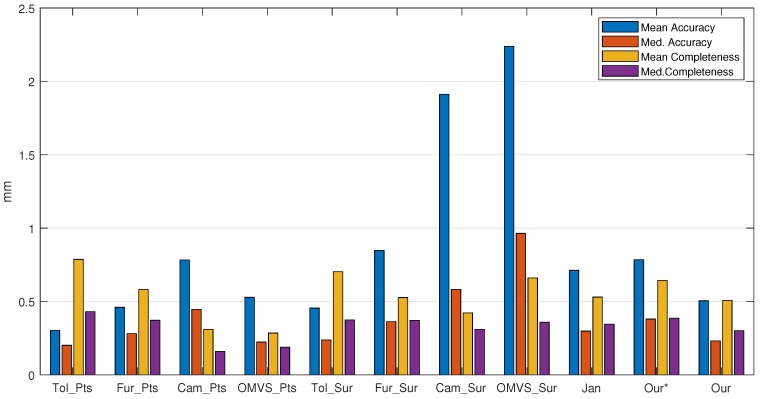
Quantitative evaluation (lower is better) of the methods on MVS dataset [2]. The abbreviations of the methods are given in Figure 10. In addition, OMVS is OpenMVS; Our∗ is the proposed method without the dense visibility technique; and ∗_Pts is the point cloud generated by method ∗.

**Figure 12:**
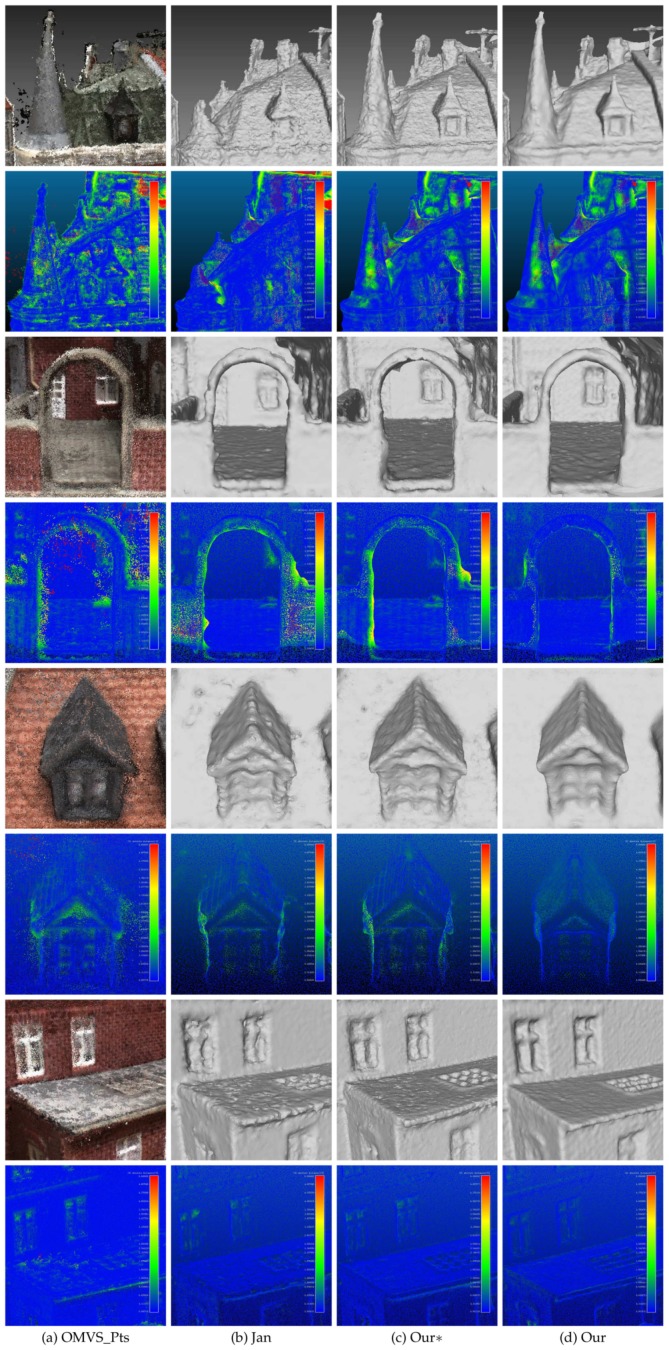
Detailed views of three methods on MVS dataset [2]. From left to right are: (**a**) the point cloud generated by OpenMVS; (**b**) the mesh of Jancosek and Pajdla [5]; (**c**) the mesh of the proposed method without the dense visibility technique; and (**d**) the mesh of the proposed method. In the even rows are the evaluation result (lower is better) of the corresponding local models in the odd rows through the method in [41]. The unit is mm for all numbers.

We first tested the proposed method on MVS dataset [2]. MVS dataset [2] contains over one hundred scenes consisting of images depicting compact objects under controlled lighting conditions. Figure 10 shows the result on the MVS dataset [2]. The reference model is in the first column. From the second column to the last column, there are the models of Tola et al. [27], Furukawa and Ponce [29], Campbell et al. [24], Jancosek and Pajdla [5] and the proposed method, respectively. The final meshes given by Tola et al. [27], Furukawa and Ponce [29] and Campbell et al. [24] were generated by Poisson surface reconstruction method [30] and trimmed, which were provided in the benchmark MVS dataset [2]; the meshes of Jancosek and Pajdla [5] were generated by OpenMVS, which contains an reimplementation of the method in [5]. Figure 10 shows that the proposed method could reconstruct complex scenes as well as regular scenes, and it had great potential for preserving scene details. Figure 11 shows the accuracy and completeness of reconstructed meshes over twenty scenes, which are scans 1–6, 9–10, 15, 21, 23–24, 29, 36, 44, 61, 110, 114, 118 and 122. The detailed information of the 3D models evaluated on MVS dataset [2] is presented in Table 2. The evaluation method is described in [2], in which accuracy is measured as the distance from the MVS reconstruction to the reference model, and the completeness is measured from the reference model to the MVS reconstruction. In addition to the evaluation of the surface, we also evaluated the point clouds generated by the methods in [24,27,29] as well as OpenMVS, with the point clouds in [24,27,29] provided by MVS dataset [2]. We can see in Figure 11 that generally the point clouds achieved better scores than the surface meshes, which complies with the results in [2]. The underlying reason could be that the mesh representation discarded points that are redundant but close to the true surface, and simultaneously fixed unexpected gaps. Comparing the result within the surface meshes, the proposed method without the dense visibility technique was not outstanding, in terms of both accuracy and completeness. By applying the dense visibility technique, the proposed method achieved the best median accuracy and median completeness, and the second best mean accuracy and mean completeness. In odd rows of Figure 12 are the local point cloud and the surface meshes of Jancosek and Pajdla [5], the proposed method without the dense visibility technique and the proposed method; in the even rows of Figure 12 are the evaluation result (lower is better) of the corresponding local models through the method in [41]. We show the ability of the three methods to preserve thin objects and object edges. In some cases, the method in [5] failed completely in reconstructing them; even the complete ones were less accurate, both visually and quantitatively, than those provided by the proposed method with or without the dense visibility technique. In addition, compared with the method in [5], the proposed method showed a tremendous capability of preserving sharp object edges.

**Figure 13:**
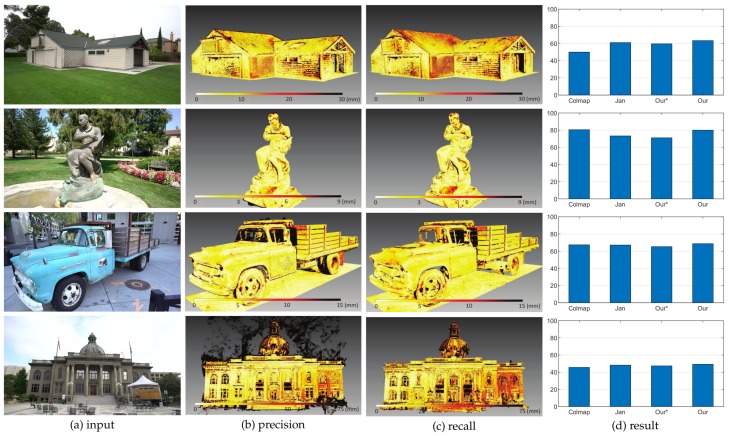
Result of the proposed method on four scenes of the training set of Tanks and Temples dataset [40]. From left to right are: (**a**) the input images; (**b**) the precision of the model generated by the proposed method; (**c**) the recall of the model generated by the proposed method; and (**d**) the evaluation result (higher is better) of the models generated by Colmap [38] and three other methods depicted in Figure 10.

**Figure 14:**
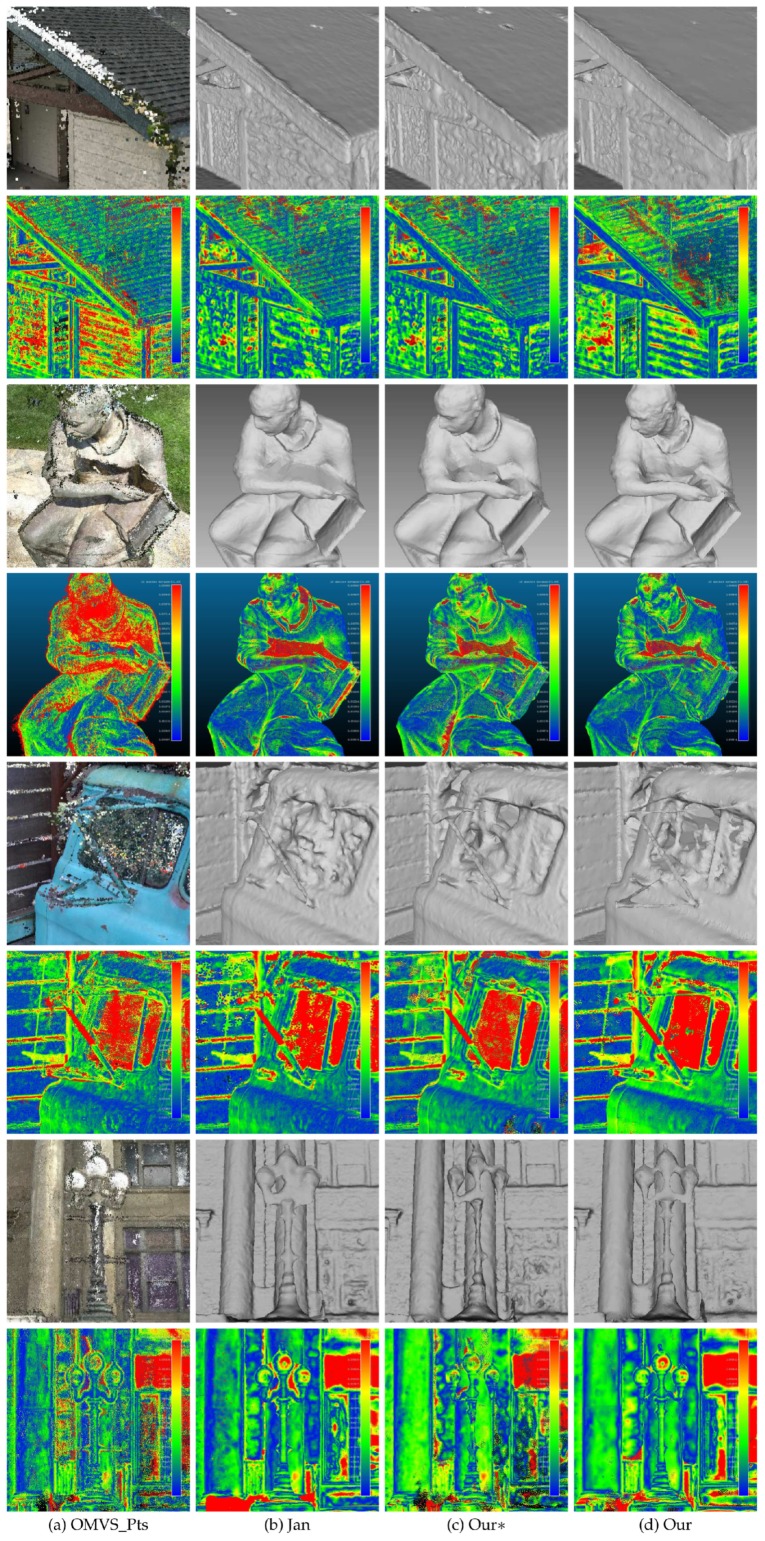
Detailed views of three methods on four scenes of the training set of Tanks and Temples dataset [40]. From left to right are: (**a**) the point cloud generated by OpenMVS; (**b**) the mesh of Jancosek and Pajdla [5]; (**c**) the mesh of the proposed method without the dense visibility technique; and (**d**) the mesh of the proposed method. In the even rows are the evaluation result (lower is better) of the corresponding local models in the odd rows through the method in [41]. The unit is m for all numbers.

**Figure 15:**
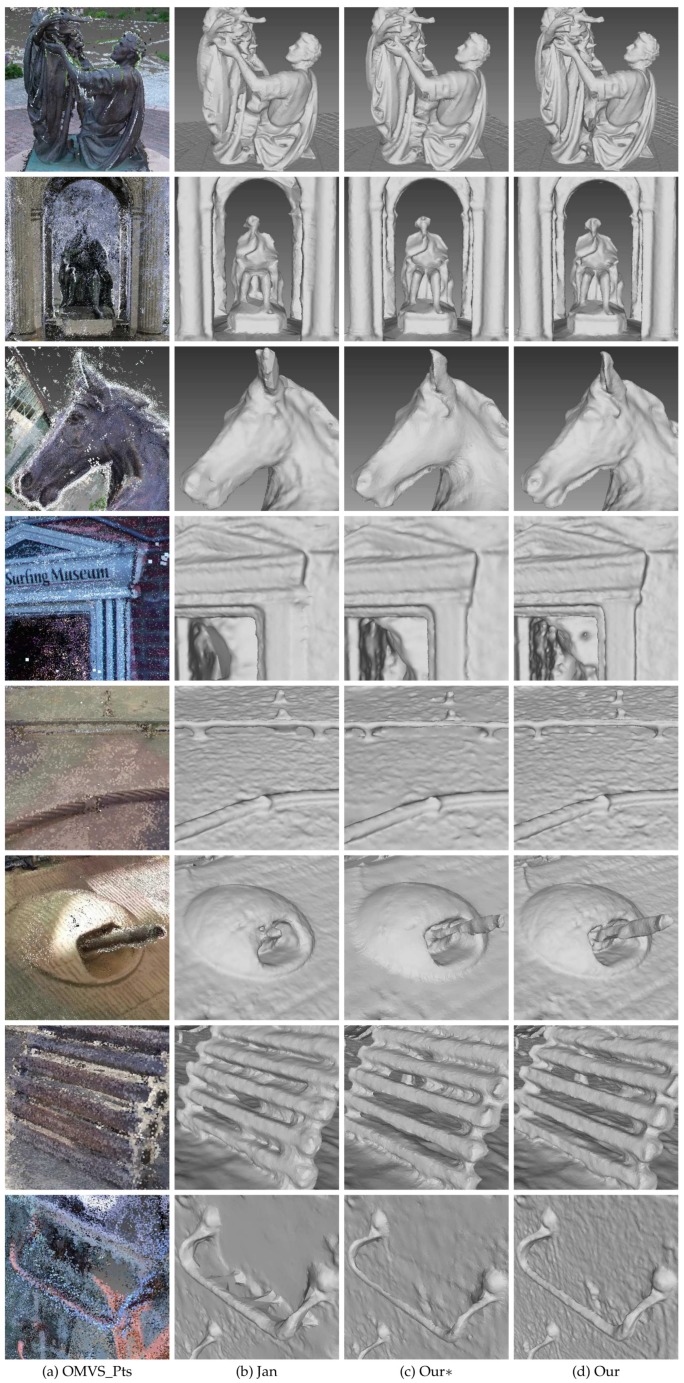
Detailed views of three methods on the intermediate set of Tanks and Temples dataset [40]. From top to bottom are Family, Francis, Horse, Lighthouse, M60, Panther, Playground and Train. From left to right are: (**a**) the point cloud generated by OpenMVS; (**b**) the mesh of Jancosek and Pajdla [5]; (**c**) the mesh of the proposed method without the dense visibility technique; and (**d**) the mesh of the proposed method.

To better visualize the differences between the models generated by Jancosek and Pajdla [5] and the proposed method, we enlarged some parts of the meshes generated by the two methods. The enlarged views are shown in Figure 12. We also tested the proposed method on benchmark Tanks and Temples dataset [40]. Scenes in Tanks and Temples dataset [40] are realistic and contain plenty of objects with different scales in both outdoor conditions and indoor environments. We evaluated the proposed method as well as two other methods on four outdoor scenes (Barn, Ignatius, Truck and Courthouse) of the training set and the eight scenes of the intermediate set of Tanks and Temples dataset [40]. Figure 13 shows the result of the proposed method on four scenes of the training set of Tanks and Temples dataset [40]. Looking at Figure 13 from left to right, we can see the input images, the precision and recall of the model generated by the proposed method and the F-scores of the models generated by Colmap [38], Jancosek and Pajdla [5], and the proposed method with and without the dense visibility technique. The evaluation method is described in [40], in which precision is measured as the distance from the MVS reconstruction to the reference model, the completeness is measured from the reference model to the MVS reconstruction, and the F-score is the harmonic mean of precision and recall with a given threshold. Table 3 presents the evaluation result of the 3D models through the method in [42]. Since the evaluation method in [40,42] takes point clouds as the input, the meshes generated by Jancosek and Pajdla [5] and the proposed method were sampled to acquire point clouds. The point clouds yielded by Colmap [38] are provided in Tanks and Temples dataset [40]. From the evaluation result of the four scenes in Figure 13 and Table 3, we concluded similarly as for the MVS dataset [2] that the proposed method outperformed the method in [5] with the dense visibility technique, while it performd slightly worse than the method in [5] without it. The proposed method performed better than the other three methods in Barn, Truck and Courthouse and rivalled Colmap [38] in Ignatius when evaluated through the method in [40], while it achieved the best result in the four scenes when evaluated through the method in [42]. Figure 14 shows the detailed views of the proposed method and the method of Jancosek and Pajdla [5] on four scenes of the training set. As in Figure 12, we also present the local models and the evaluation result using the method in [41] in odd rows and even rows, respectively. In Figure 14, Jancosek and Pajdla’s method [5] inaccurately reconstructed the edge of the roof, wrongly fixed the gap between the arm and the chest of the statue, and failed to reconstruct the rearview mirror of the truck and the light stand of the lamp, while the proposed method performed well in these parts. It is noteworthy that the proposed method also had a great ability of deducing the close form of the real surface when there were no supportive points due to the occlusion, a common phenomenon in 3D reconstruction. A good example is the arm and the chest of the statue in Figure 14. We can see that the proposed method fixed the vacant part of the chest reasonably, even though it was occluded by the arm of the statue, while the method in [5] gave a less satisfactory solution. The underlying reason is that, in the proposed visibility models, the end of a line of sight lies in the tetrahedra right behind the 3D point, which is good for separating small scale objects from other objects. Table 4 and Figure 15 present the result of the proposed method on the intermediate set of Tanks and Temples dataset [40]. The detailed information of the 3D models evaluated on the intermediate set of Tanks and Temples dataset [40] is presented in Table 5. In Table 4, we can see that the result of the proposed method and that in [5] are not outstanding. However, compared to the evaluation result of the point clouds of OpenMVG + OpenMVS, the result of both the proposed method and the method in [5] achieves a substantial boost in all scenes except M60. The underlying reason could be that the two methods simultaneously filtered most noise points and fixed unexpected gaps during the surface reconstruction process. These two behaviors had opposite effects on the evaluation result, since the former one increased the F-score while the latter one decreased it. In Table 4, we can also find that generally with the dense visibility technique the proposed method outperformed the method in [5], while without the dense visibility technique it performed worse than the method in [5]. Figure 15 shows the detailed views of the proposed method and the method of Jancosek and Pajdla [5] on eight scenes of the intermediate set. Table 4 shows the F-scores of several public methods and the proposed method on eight scenes of the intermediate set. In dealing with the thin objects such as the human legs in Francis, the horse ear in Horse, the wire in M60, the barrel in Panther, the bar in Playground and the handle in Train, Jancosek and Pajdla’s method [5] either failed to reconstruct it or wrongly fixed the gaps, while the proposed method performed well in these parts. Compared to the meshes of the method in [5], the proposed method also kept distinguishing object edges such as the cloth folds in Family, the horse mouth in Horse and the doorframe in Lighthouse. Therefore, we can infer that the proposed method has a strong ability to preserve the scene details.

## 8. Conclusions

In this paper, we present a new surface reconstruction method. The proposed method is designed to preserve scene details while keeping the ability to filter noise. To make the proposed method efficient to filter out noise and to select true surface points, we introduce a new visibility model with error tolerance and adaptive end weights. Along with the proposed visibility model, a new likelihood energy term is added to the total energy of the binary labeling problem to promote the robustness of the proposed method to noise. Moreover, we further improve the performance of the proposed method with the dense visibility technique, which avoids the error introduced in the point cloud generation process and provide denser visibility information. We tested the proposed method on two publicly available benchmark datasets. Experimental results on different datasets show that the proposed method rivalled the state-of-the-art methods in terms of accuracy and completeness, while it could preserve scene details such as thin objects and sharp edges. Our future work will consist in segmenting the model and adding semantic knowledge to each part of the model.

## Figures and Tables

**Table 1:** Symbols used in this work.

Symbol	Meaning
*v*	line of sight
*c*	camera center (a 3D point)
*p*	3D point
*T*	tetrahedron
$l_{T}$	label of tetrahedron *T*
$D(l_{T})$	unary energy of the label assignment of tetrahedron *T*
$W(l_{T_{i}},l_{T_{j}})$	pair-wise energy of the label assignments of two adjacent tetrahedra
$\alpha_{v}$	weight of a line of sight *v*
$N_{v}$	amount of tetrahedra intersected with a line of sight *v*
*d*	distance between point *p* and the intersecting point of a segment and a facet
σ	scale factor
*r*	the radius of the circumsphere of the end tetrahedron
$U_{out}\left(T\right)$	energy of tetrahedron *T* being labeled as outside
$U_{in}\left(T\right)$	energy of tetrahedron *T* being labeled as inside
f(T)	free-space support of tetrahedron *T*
β	constant for transferring f(T)
$\lambda ,\lambda_{vis},\lambda_{like},\lambda_{qual}$	balance factor
$E,E_{vis},E_{like},E_{qual},E_{vis}^{typical}$	energy
$w_{f}$	weight of a facet *f*
ϕ,ψ	angle

**Table 2:** Information of the 3D models evaluated on MVS dataset [2]. ∗_Pts is the number of points in a 3D point cloud. ∗_Vtx and ∗_Fcs are the number of vertices and facets of a 3D mesh, respectively. The unit of all numbers is million.

SceneID	1	2	3	4	5	6	9	10	15	21	23	24	29	36	44	61	110	114	118	122
Tol_Pts	1.0	1.1	0.9	0.7	0.9	1.0	1.0	0.7	1.0	1.0	1.1	0.8	0.7	1.1	0.9	0.7	0.7	1.2	1.0	0.9
Tol_Vtx	2.1	2.2	2.1	1.8	1.9	2.3	2.6	1.7	2.3	2.1	2.3	2.4	1.1	2.0	1.5	1.6	1.6	2.1	2.1	1.8
Tol_Fcs	4.2	4.4	4.2	3.5	3.7	4.6	5.2	3.3	4.7	4.3	4.5	4.8	2.1	4.1	3.1	3.2	3.2	4.2	4.2	3.5
Fur_Pts	2.3	2.6	2.5	2.2	2.2	2.4	2.4	1.9	2.5	3.0	3.1	2.5	2.3	2.7	2.7	1.6	2.2	2.6	2.6	2.4
Fur_Vtx	1.1	1.1	1.2	0.8	0.8	0.7	1.0	0.7	2.5	2.9	2.8	1.0	2.1	1.9	1.7	0.9	1.8	1.5	1.7	1.4
Fur_Fcs	2.2	2.2	2.4	1.6	1.6	1.5	2.0	1.5	4.9	5.8	5.5	1.9	4.2	3.8	3.4	1.7	3.6	2.9	3.3	2.7
Cam_Pts	23.6	29.6	22.2	20.8	20.2	23.6	19.8	13.0	22.0	24.0	29.5	20.2	16.5	29.5	20.2	7.6	19.9	26.1	30.2	21.7
Cam_Vtx	4.2	4.6	8.1	4.8	6.8	6.7	16.0	2.6	12.0	8.9	4.1	2.6	3.3	3.2	5.1	3.7	6.3	5.1	31.2	6.1
Cam_Fcs	8.5	9.2	16.3	9.5	13.5	13.4	32.0	5.1	24.0	17.8	8.2	5.2	6.6	6.3	10.2	7.3	12.5	10.2	62.4	12.1
OMVS_Pts	11.8	11.0	12.2	10.1	11.8	10.9	9.1	8.3	9.2	10.1	12.2	9.0	7.8	11.3	9.8	8.9	8.0	13.1	8.8	8.4
Jan_Vtx	0.6	0.6	0.7	0.5	0.6	0.6	0.6	0.5	0.6	0.8	0.7	0.6	0.6	0.7	0.7	0.5	0.5	0.7	0.6	0.6
Jan_Fcs	1.3	1.2	1.4	1.1	1.2	1.2	1.2	1.0	1.3	1.6	1.4	1.2	1.2	1.5	1.5	0.9	1.0	1.3	1.2	1.1
Our*_Vtx	1.2	1.1	1.1	1.0	0.9	1.0	1.0	0.9	1.1	1.3	1.3	1.2	1.0	1.3	1.1	0.7	1.0	1.1	0.9	1.0
Our*_Fcs	2.5	2.3	2.2	2.0	1.8	2.0	2.0	1.9	2.2	2.6	2.6	2.4	2.0	2.6	2.3	1.5	1.9	2.2	1.8	1.9
Our_Vtx	1.6	1.6	1.4	1.0	1.2	1.4	1.3	1.2	1.5	1.5	1.7	1.3	1.3	1.3	1.2	0.7	1.0	1.5	1.2	1.1
Our_Fcs	3.3	3.2	2.9	2.0	2.5	2.9	2.6	2.4	3.0	3.1	3.4	2.6	2.7	2.6	2.5	1.4	2.0	3.1	2.4	2.3

**Table 3:** Evaluation result of the 3D models evaluated on the training set of Tanks and Temples dataset [40] through the method in [42]. M stands for million. Precision is expressed as a proportion 1:k, where k is the size of the scene divided by the standard error. The unit of all other numbers is mm.

**Type**	**Barn**					**Ignatius**				
	**Colmap**	**OMVS_Pts**	**Jan**	Our*	**Our**	**Colmap**	**OMVS_Pts**	**Jan**	Our*	**Our**
Pts	6.2M	35.4M	6.3M	5.8M	6.7M	1.3M	13.1M	3.5M	2.9M	3.3M
mean	19.24	17.70	10.44	11.14	10.23	2.66	3.55	2.51	2.90	2.14
95.5%<	59.93	34.75	28.17	29.42	26.04	7.96	12.15	6.33	7.69	5.15
99.7%<	221.83	181.46	118.49	120.25	113.45	39.18	35.91	38.55	38.49	34.38
Precision	1:800	1:1000	1:1400	1:1400	1:1500	1:500	1:600	1:800	1:800	1:900
**Type**	**Courthouse**					**Truck**				
	**Colmap**	**OMVS_Pts**	**Jan**	Our*	**Our**	**Colmap**	**OMVS_Pts**	**Jan**	Our*	**Our**
Pts	17.3M	63.4M	14.4M	13.7M	15.0M	3.8M	22.5M	3.7M	3.0M	3.5M
mean	97.56	247.94	93.15	96.84	91.88	8.07	7.29	6.65	7.13	6.47
95.5%<	315.51	597.98	302.36	311.29	294.31	24.67	23.62	21.48	23.87	21.46
99.7%<	2261.96	5108.95	2086.73	2117.38	2029.45	154.56	174.76	147.83	151.72	143.58
Precision	1:300	1:200	1:400	1:400	1:400	1:400	1:400	1:600	1:600	1:700

**Table 4:** Leaderboard ${}^{1}$ of the methods and the result of the proposed method with respect to F-score on the intermediate set of Tanks and Temples dataset [40].

Method	Family	Francis	Horse	Lighthouse	M60	Panther	Playground	Train	Mean
PMVSNet	70.04	44.64	40.22	**65.20**	**55.08**	**55.17**	**60.37**	**54.29**	55.62
Altizure-HKUST	**74.60**	**61.30**	38.48	61.48	54.93	53.32	56.21	49.47	**56.22**
ACMH	69.99	49.45	**45.12**	59.04	52.64	52.37	58.34	51.61	54.82
Dense R-MVSNet	73.01	54.46	43.42	43.88	46.80	46.69	50.87	45.25	50.55
R-MVSNet	69.96	46.65	32.59	42.95	51.88	48.80	52.00	42.38	48.40
i23dMVS4	56.64	33.75	28.40	48.42	39.23	44.87	48.34	37.88	42.19
MVSNet	55.99	28.55	25.07	50.79	53.96	50.86	47.90	34.69	43.48
COLMAP	50.41	22.25	25.63	56.43	44.83	46.97	48.53	42.04	42.14
Pix4D	64.45	31.91	26.43	54.41	50.58	35.37	47.78	34.96	43.24
i23dMVS_3	56.21	33.14	28.92	47.74	40.29	44.20	46.93	37.66	41.89
OpenMVG + OpenMVS	58.86	32.59	26.25	43.12	44.73	46.85	45.97	35.27	41.71
OpenMVG + MVE	49.91	28.19	20.75	43.35	44.51	44.76	36.58	35.95	38.00
OpenMVG + SMVS	31.93	19.92	15.02	39.38	36.51	41.61	35.89	25.12	30.67
Theia-I + OpenMVS	48.11	19.38	20.66	30.02	30.37	30.79	23.65	20.46	27.93
OpenMVG + PMVS	41.03	17.70	12.83	36.68	35.93	33.20	31.78	28.10	29.66
Jan	62.69	47.44	34.52	57.94	38.67	47.06	55.26	39.90	47.94
Our*	62.46	46.68	32.61	57.66	33.66	44.25	52.40	38.25	46.00
Our	65.21	49.41	35.41	59.04	37.57	47.85	56.77	41.28	49.07

${}^{1}$https://www.tanksandtemples.org/leaderboard/.

**Table 5:** Information of the 3D models evaluated on the intermediate set of Tanks and Temples dataset [40]. ∗_Pts is the number of points in a 3D point cloud. ∗_Vtx and ∗_Fcs are the number of vertices and facets of a 3D mesh, respectively. The unit of all numbers is million.

Scene	Family	Francis	Horse	Lighthouse	M60	Panther	Playground	Train
OMVS_Pts	12.0	17.8	9.0	28.4	24.8	26.0	28.6	31.9
Jan_Vtx	2.0	1.9	1.5	2.8	5.1	4.1	5.7	4.8
Jan_Fcs	4.1	3.7	3.0	5.6	10.1	8.3	11.3	9.6
Our∗_Vtx	1.6	1.2	1.2	1.7	3.6	2.9	4.1	3.2
Our∗_Fcs	3.2	2.4	2.3	3.5	7.2	5.8	8.2	6.5
Our_Vtx	2.5	2.7	2.1	3.3	5.3	5.4	6.9	6.3
Our_Fcs	5.1	5.4	4.2	6.6	10.6	10.8	13.9	12.5
